# Dosimetric characterization of GMS BT‐125‐1 ^125^I radioactive seed with Monte Carlo simulations and experimental measurement

**DOI:** 10.1002/acm2.12173

**Published:** 2017-09-14

**Authors:** Nan Zhao, Ruijie Yang, Li Ren, Yi Fan, Junli Li, Jianguo Zhang

**Affiliations:** ^1^ Department of Radiation Oncology Peking University Third Hospital Beijing China; ^2^ Key Laboratory of High Energy Radiation Imaging Fundamental Science Key Laboratory of Particle & Radiation Imaging (Tsinghua University) Ministry of Education Department of Engineering Physics Tsinghua University Beijing China; ^3^ Department of Oral and Maxillofacial Surgery Peking University School and Hospital of Stomatology Beijing China

**Keywords:** dosimetry, I‐125 radioactive seed, Monte Carlo simulation, thermoluminescent dosimeters

## Abstract

**Purpose:**

To investigate the dosimetric characteristics of the new GMS BT‐125‐1 ^125^I radioactive seed, including dose rate constant, radial dose functions, and anisotropy functions.

**Methods:**

Dosimetric parameters of GMS BT‐125‐1 ^125^I seed including dose rate constant, radial dose functions, and anisotropy functions were calculated using the Monte Carlo code of MCNP5, and measured with thermoluminescent dosimeters (TLDs). The results were compared with those of PharmaSeed BT‐125‐1, PharmaSeed BT‐125‐2 ^125^I, and model 6711 ^125^I seeds.

**Results:**

The dose rate constant of GMS BT‐125‐1 ^125^I seed was 0.959 $cGy\cdot h^{-1}\cdot U^{-1}$, with the difference of 0.94%, 0.83%, and 0.73% compared with the PharmaSeed BT‐125‐1 ^125^I seed, PharmaSeed BT‐125‐2 ^125^I seed, and Model 6711 ^125^I seed, respectively. For radial dose function, the differences between the Monte Carlo and the experimental *g*(*r*) results were mostly within 10%. Monte Carlo results of *g*(*r*) for GMS BT‐125‐1 ^125^I seed were found in agreement (within 3.3%) with corresponding results for the PharmaSeed BT‐125‐2 ^125^I seed. The largest differences were 8.1% and 6.2% compared with PharmaSeed BT‐125‐1 ^125^I seed and model 6711 ^125^I seed, respectively. For anisotropy function, the difference between GMS BT‐125‐1 ^125^I seed and PharmaSeed BT‐125‐2 ^125^I seed was typically <10%.

**Conclusions:**

The measured dose rate constant, radial dose functions, and two‐dimensional anisotropy functions for the GMS BT‐125‐1 ^125^I seed showed good agreement with the Monte Carlo results. The dose rate constant of the GMS BT‐125‐1 ^125^I seed is similar to that of the PharmaSeed BT‐125‐1 ^125^I seed, the PharmaSeed BT‐125‐2 ^125^I seed, and the model 6711 ^125^I seed. For radial dose functions and two‐dimensional anisotropy functions, the GMS BT‐125‐1 ^125^I seed is similar to the PharmaSeed BT‐125‐2 ^125^I seed but different from the PharmaSeed BT‐125‐1 ^125^I seed and the model 6711 ^125^I seed.

## 1 INTRODUCTION

Brachytherapy, using permanently implanted seeds, has become widely accepted for low‐risk prostate cancer. It has been proven to be as effective as surgery or external beam radiotherapy.^1^ In China, permanent seed implants have also been used in the treatment of primary and recurrent tumors of the head and neck, lungs, liver, pancreas, rectal, gynecological, and soft tissue.^2^, ^3^, ^4^, ^5^, ^6^, ^7^, ^8^, ^9^ Tens of thousands of patients were treated in hundreds of hospitals. Both the clinical outcome and side effects of seed implantation depend on the dose to the tumor and organs at risk (OARs). Zelefsky et al^1^ found that patients, where the ^125^I dose to 90% of the prostate (D_90_) was > or = 130 Gy, the 8‐year prostate‐specific antigen (PSA) relapse‐free survival was 93% compared with 76% for those with lower D_90_ dose levels (*P* < 0.001). Stock et al^10^ found that the optimal dosimetry of ^125^I seed implantation is D_90_ between 140 and 180 Gy for prostate cancer. The biochemical failure increases when D_90_ is less than 140 Gy, and the long‐term urinary symptoms increase when D_90_ is more than 180 Gy. So, the accuracy of the dosimetry plays an important role in the outcome of brachytherapy. A treatment planning system is widely used for seed implantation for preoperative dose calculation, postoperative dose verification, and real‐time dose verification.^11^, ^12^, ^13^, ^14^, ^15^ One of the most important factors for the accuracy of the dose calculations is the accurate dosimetric parameter of each type of ^125^I seed used for implantation.

The GMS BT‐125‐1^125^I seed, manufactured by the global medical solutions (GMS) Pharmaceutical Company, was widely used in clinical practice. But, there are no dosimetric parameters of it for the dose calculation. Instead, the dosimetric parameters of the Model 6711^125^I seed were used. But the length of the silver rod in the GMS BT‐125‐1 ^125^I seed is 3.25 mm, which is different from that in the Model 6711 ^125^I seed that is 3.00 mm. Although the structure of the GMS BT‐125‐1 ^125^I seed is similar to that of the Model 6711 ^125^I seed, the different length of core markers cause different dose distributions of radioactive seeds.^16^ Both GMS BT‐125‐1 ^125^I seed and PharmaSeed BT‐125‐1 ^125^I seed have a wire core with the length of 3.25 mm that was surrounded by 0.5 μm layer absorbed with ^125^I. However, a silver rod is used as the wire core in GMS BT‐125‐1 ^125^I seed, while a palladium rod is used in the PharmaSeed BT‐125‐1 ^125^I seed. So, the dosimetric parameters of the PharmaSeed BT‐125‐1 ^125^I seed cannot be used as a substitute for GMS BT‐125‐1^125^I seed.

AAPM recommends dosimetry characterization of new low‐energy seeds including dose rate constant Λ, radial dose function, *g*(*r*), and two‐dimensional anisotropy function F(r,θ) before clinical application using both experimental and Monte Carlo methods.^16^, ^17^ TG‐43 U1 recommends the updated low‐energy photon cross‐sections in brachytherapy source dosimetry. The photon cross‐sections were updated in the Monte Carlo N‐Particle Transport Code 5 (MCNP5).^16^, ^18^

The aim of this study is to characterize the dosimetric parameters of a new GMS BT‐125‐1 ^125^I seed using both the experimental measurement and Monte Carlo simulation with MCNP5 code.

## 2 METHODS

### 2.A Radioactive seeds

**Figure 1:**
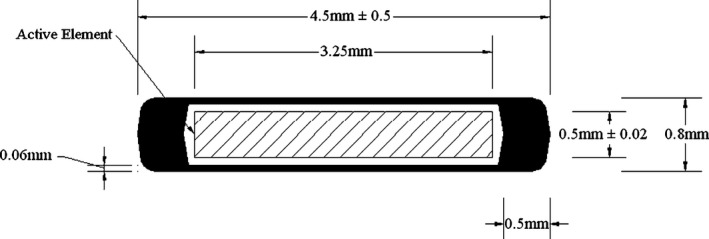
Schematic diagram of the GMS BT‐125‐1 ^125^I seed.

The GMS BT‐125‐1 ^125^I radioactive seed was manufactured by global medical solutions (GMS) Pharmaceutical Company in Shanghai, China. The structure and dimensions of the GMS BT‐125‐1 ^125^I seed are presented in Fig. 1. The core of the radioactive seed consists of a cylindrical silver marker (10.53 g/cm^3^), with the length of 3.25 mm and diameter of 0.50 ± 0.02 mm. The silver marker is coated with radioactive ^125^I with the thickness of 0.5 μm. The length of radioactive ^125^I is 3.25 mm. The radioactive ^125^I is encapsulated in a hollow titanium (Ti) tube (4.5 g/cm^3^), which is 4.5 ± 0.5 mm in length and 0.8 mm in external diameter, with a wall thickness of 0.06 mm after welding. The tube is sealed by laser welding of hemispherically shaped ends of a 0.5 mm radius. The conical angle of the end cap is 180°. The Model 6711 ^125^I seed consists of a 4.5 mm long welded titanium capsule with a thickness of 0.05 mm. The capsule contains a 3.0 mm long silver rod that was surrounded with absorbed ^125^I.^16^

### 2.B Monte Carlo simulation

The Monte Carlo simulation is the most commonly used method to determine the dosimetric parameters of low or high‐energy radioactive sources.^16^, ^18^, ^19^, ^20^ We used a general Monte Carlo code of MCNP5 developed by Los Alamos National Laboratory in this study.^21^ The photon cross‐section library used in this code is MCNPLIB04, and the electron cross‐section library is EL03. We performed the Monte Carlo simulation of the Model 6711 ^125^I seed using MCNP5 to compare with the results provided by TG‐43U1 for the benchmark of Monte Carlo simulation.

### 2.C Experiment set

The accurate geometry and materials of the GMS BT‐125‐1 ^125^I seed were described above. The simulated seed was positioned within the center of a water sphere with the diameter of 30 cm. The ^125^I photon spectrum was taken from AAPM TG‐43U1, which was based on the National Nuclear Data Center spectrum.^22^ The spectrum consisted of five energies in the following abundance: 27.2 keV (27.5%), 27.5 keV (51.3%), 31.0 keV (13.7%), 31.7 keV (3.0%), and 35.5 keV (4.5%). According to AAPM Task Group 43,^17^ the dose rate constant is defined as the ratio of the dose rate at a reference distance per unit kerma strength: Λ = *D*(*r*_0,_ θ_0_)/*S*_*k*_. The dose rate was determined in water at a distance of 1 cm from the ^125^I seed. For the determination of Λ, the seed was placed in the center of a vacuum sphere with a 15 cm radius. In this simulation, an energy cutoff of δ = 5 keV was used to suppress the characteristic x‐rays generated in the Ti encapsulation as recommended by TG43U1.^16^ A 1 mm^3^ scoring three‐dimensional voxel was located 10 cm from the seed perpendicular axis according to Meigooni et al's study.^24^ To determine the air kerma in the cell, the MCNP *F4 tally (MeV/cm^2^) was used for determining the energy fluence in the cell. To determine the air kerma, the MCNP's DE/DF card was used. The simulations were performed with 1 × 10^9^ histories. The dimensional sizes of the water phantoms and holes simulated in the Monte Carlo simulation were the same with those in the measurement. The conversion factors that convert the results from PMMA phantom to water phantom were obtained using the Monte Carlo simulation as well. The dose rate constant, radial dose function, and anisotropy function were determined in PMMA phantom and liquid water using the Monte Carlo simulation, respectively. The conversion factor from PMMA to liquid water was derived as the ratio of the numbers in liquid water to the numbers in PMMA phantom.^23^ The geometry function was calculated using the AAPM Task Group 43 approximation for a line source [eq. (1)].

(1) G(r,θ)=β/Lrsinθ

The radial dose function was determined by calculating the dose rate in water at distances of 0.5, 0.7, and 1.0–10.0 cm with an increment of 0.5 cm from the center of the source using F8 tally. According to the TG43 report, the Monte Carlo simulated radial dose functions were fit to a fifth order polynomial, $g\left(r\right)=a_{0}+a_{1}r+a_{2}r^{2}+a_{3}r^{3}+a_{4}r^{4}+a_{5}r^{5}$. As described above, the MCNP *F8 tally was used to score energy fluence (MeV) at different distances. The tally voxel size is constant across the distance. Primary particles (5 × 10^9^) were followed resulting in statistical uncertainty ranging from 0.5% to 2.5% at 0.5 and 10 cm, respectively. The dose rate was normalized to unity at the distance of *r*_0_ = 1.0 cm, and the radial dose function was calculated according to eq. (2) using the geometry function.

(2) $$g\left(r\right)=\frac{D\left(r,\theta_{0}\right)G\left(r_{0},\theta_{0}\right)}{D\left(r_{0},\theta_{0}\right)G\left(r,\theta_{0}\right)}$$

The two‐dimensional anisotropy function [eq. (3)] was determined by scoring the dose rate in water in spherical volumes located at distance of 0.5, 0.7, 1.0, 1.5, 2.0, 3.0, 4.0, 5.0, 6.0, and 7.0 cm from the center of the source using F8 tally. At a distance of 0.5 cm, the interval of an adjacent scoring point is 20°, while at other distances, the interval is 10°. Due to the small size of each scoring volume, it was necessary to follow 5 × 10^9^ primary particles to achieve statistically meaningful results. Statistical uncertainties ranged from 0.5% to 1.1% at 0.5 and 7 cm, respectively. At each radial distance, dose rate was normalized to unity at an angle of 90°.

(3) $$F\left(r,\theta \right)=\frac{D\left(r,\theta \right)G\left(r,\theta_{0}\right)}{D\left(r,\theta_{0}\right)G\left(r,\theta \right)}$$

### 2.D Thermoluminescence dosimetry

A total of 10 TLDs were irradiated using 48 keV x‐ray in National Institute of Metrology of China. According to the study conducted by Saez‐Vergara et al,^24^ the relative energy response of GR‐200 for 33 and 48 keV in PMMA were 1.14 and 1.17, respectively. The GR‐200 TLDs have the same composition and characteristics with the TLDs that we used. So, we used the 48 keV x‐ray to calibrate the TLDs.

**Figure 2:**
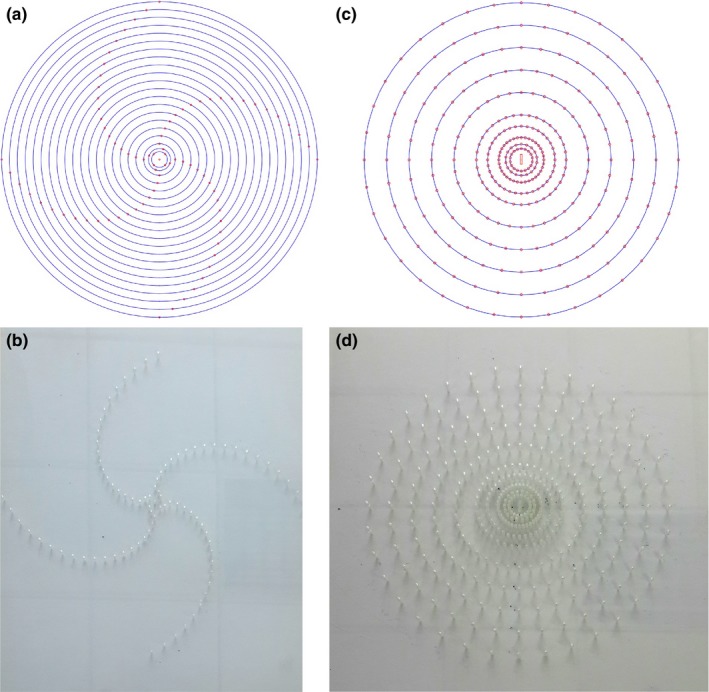
(a) and (b) Phantom I was used for the determination of the dose rate constant and radial dose function for the GMS BT‐125‐1 ^125^I seed. The seed lies in the middle of the phantom. Its long axis is perpendicular to the slab plane. The dosimeters lie at radial distances of 0.5, 0.7, and 1–10 cm with an increment of 0.5 cm and 5°. (c) and (d) Phantom II was used for the measurement of the two‐dimensional anisotropy function of the GMS BT‐125‐1 ^125^I seed. The seed lies in the middle of the phantom. It is placed with its long axis parallel to the central plane of the slabs. The dosimeters lie at radial distances of *r* = 0.5 cm, 0.7, 1, 1.5, 2, 3, 4, 5, 6, and 7 cm from the seed center, and polar angles θ ranging from 0° to 350° in 10° increments with respect to the seed long axis, except the distance of 0.5 cm, which has the increment of 20°.

Before measuring the dosimetric parameters, the source strength of the seeds was independently measured using the HDR 1000 Plus Well Chamber (Standard imaging lnc., USA). Dose distributions around the GMS BT‐125‐1 ^125^I seed were measured in a pure polymethyl methacrylate (PMMA) phantom using TLD2000 LiF (Mg, Cu, P) thermoluminescent dosimeters. Phantom I was used for the determination of the dose rate constant and radial dose function [Fig. 2(a) and Fig. 2(b)]. It consisted of PMMA slabs that comprised a cubic phantom of 30 × 30 × 30 cm^3^. The density of the pure PMMA (C_5_O_2_H_8_) is 1.18 g/cm^3^. Holes were drilled in the central phantom slab to accommodate the TLD rods so that their long axes were perpendicular to the slab plane and thus parallel to the source long axis, with the centers of the dosimeters and the center of the source being in the same plane. These holes were at radial distances of 0.5, 0.7, and 1–10 cm with an increment of 0.5 cm and 5° so that there would be no shadowing effects for measurements. Phantom II was used for the measurement of the two‐dimensional anisotropy function of the GMS BT‐125‐1 ^125^I seed [Fig. 2(b) and Fig. 2(d)]. It has the same geometry and dimensions as the phantom I but differs in the configuration of the source in that the seed is placed with its long axis parallel to the central plane of the slabs. The cylindrical TLD is 6 mm in length, and 1 mm in diameter. The TLD dosimeters were placed with their long axes perpendicular to the central plane of the slabs, and also vertical to the longitudinal axis of the seed. The dosimeters lie at radial distances of *r* = 0.5, 0.7, 1, 1.5, 2, 3, 4, 5, 6, and 7 cm from the seed center, and polar angles θ ranging from 0° to 350° in 10° increment with respect to the seed's long axis except the distance of 0.5 cm in 20° increment. Measurements were performed with all holes containing a TLD dosimeter since it was found that for the experimental two‐dimensional anisotropy function determination at a specific (*r*, θ) point, shadowing effects due to the dosimeters that lie at the same polar angle and radial distances mentioned above do not affect results. This is due to the definition of the two‐dimensional anisotropy function that normalizes dose rate at a particular (*r*, θ) point to the dose rate at the corresponding point along the transverse source bisector, (*r*, 90°).^16^ Therefore, since shadowing was found similar to any polar angle for the same radial distance, the overall effect is canceled out in the calculation of the two‐dimensional anisotropy function. The results of one particular angle and its supplementary angle of 180° were averaged as the final results.

The dosimetric parameters in the TG43U1 were evaluated in liquid water.^16^ So, the TLD measured results in PMMA were corrected to those with liquid water as phantom material. The corresponding correction factor, *P*_*phant*_(*r*, θ), was calculated by the following ratio: $P_{phant}\left(r,\theta \right)={\left[\frac{D_{w}^{w}\left(r,\theta \right)}{D_{w}^{PMMA}\left(r,\theta \right)}\right]}_{125_{I}}$, where $D_{w}^{w}\left(r,\theta \right)$ and $\frac{D_{w}^{w}\left(r,\theta \right)}{D_{w}^{PMMA}\left(r,\theta \right)}$ were the dose to the detector in liquid water and PMMA phantom material using the Monte Carlo simulation, respectively, for the ^125^I seed.

Immediately before exposure, all dosimeters were annealed for 10 min at 240°C and then rapidly cooled down to room temperature.^25^ A Harshaw Model 3500 automatic reader (Thermo Fisher Scientific, Inc., Waltham, MA, USA) was used for reading the dosimeters. The reader was purged with nitrogen, and the following cycle was used: heating at 135°C for 8 s, followed by heating at a constant rate to 240°C in 20 s. Data were collected during the second phase of the readout cycle.^25^ Prior to performing the experimental reading, 2–3 unexposed detectors were read to stabilize the readers. TLD readings were converted to absolute dose rate before the calculation of all the dosimetric parameters.

Six different seeds were used in all measurements for calculating the dosimetric parameters. The mean of the measurements was reported in this study.

### 2.E Uncertainty of the study

Uncertainty includes type A error that is random error and type B error that is the systematic error.^16^, ^20^ For the Monte Carlo simulation, type A errors included the statistical errors and errors inherent to the Monte Carlo technique. Type B errors included the uncertainty of the underlying cross‐sections. The uncertainty of the position of the silver markers inside the seed and the uncertainty in determining geometry structures of the seed were also considered as seed geometry uncertainty in the Monte Carlo simulation.

The uncertainties associated with our TLD measurements were estimated by following the guidelines of the AAPM TG‐138 and TG‐43U1 reports.^16^, ^26^ The uncertainty associated with TLD‐seed relative positioning should take into account the 0.05 mm machining tolerance of the PMMA phantom reported by the manufacturer. The uncertainty of the strength of the seeds was the difference between the labeled strength and the measured strength using the well‐type ionization chamber. The experimental results of radial dose and two‐dimensional anisotropy function were not affected by the TLD calibration factor due to their relative number compared with the result located 1 cm from the seed.

The uncertainty of the dose rate constant is the square root of the sum of the squares of the relative dose rate uncertainties at the reference position and air‐kerma strength, $\%u_{\Lambda }=\sqrt{\%{u_{D(r_{0},\theta_{0})}}^{2}+\%{u_{s_{k}}}^{2}}$, $\%u_{D(r_{0},\theta_{0})}$, and $\%u_{s_{k}}$ represent the uncertainty of the relative dose rate at the reference point and air‐kerma strength. For the TLD measured dose rate constant, a total relative standard uncertainty was estimated to be 7.6%. As listed in Table 1, the estimation took into account the uncertainty associated with the seed strength (3.0%), seed and TLD positioning in the PMMA phantom (3.5%), TLD dose calibration (2.6%), PMMA‐to‐water conversion (3.0%), and statistical variations of TLD readout in repetitive measurements (4.5%). The uncertainty of the measured radial dose function depended on the relative uncertainty of dose rate measurements at both the reference position and the point of interest on the transverse plane. The uncertainties of the radial dose function were primarily due to the uncertainties in the seed‐to‐TLD distance, repetitive TLD measurements, and PMMA‐to‐water conversion. For the dose rate constant simulated by the Monte Carlo, a total relative standard uncertainty was estimated to be 5.4%. As listed in Table 2, the estimation took into account the uncertainty associated with the MC Statistics including the uncertainty of air‐kerma strength (3.2%) and uncertainty of dose deposition (0.5%), cross‐sections (1.5%), seed geometry (2.0%), seed spectrum (0.2%), and seed/TLD positioning uncertainty (3.5%). The relative standard uncertainty for the Monte Carlo calculation was estimated from the relative uncertainties associated with the dose rates calculated in water as a function of radial distance, which increased from 0.5% at 1 cm to 0.6%, 0.8%, and 1.1% at 3, 5, and 7 cm, respectively.

**Table 1:** Calculated uncertainties associated with the derivation of dose rate constant using experimental results

Component	Relative standard uncertainty (%)	
	Type A	Type B
Seed strength		3.0
TLD source position		3.5
TLD dose calibration		2.6
PMMA‐to‐water conversion		3.0
Repetitive measurements	4.5	
Total standard uncertainty	7.6	

**Table 2:** Calculated uncertainties associated with the derivation of dose rate constant using the Monte Carlo simulation

Component	Relative standard uncertainty (%)	
	Type A	Type B
MC Statistics	3.2	
Seed/TLD positioning uncertainty		3.5
Cross‐sections		1.5
Seed geometry		2.0
Source spectrum		0.2
Dose deposition	0.5	
Total standard MC uncertainty	5.4	

## 3 RESULTS

**Figure 3:**
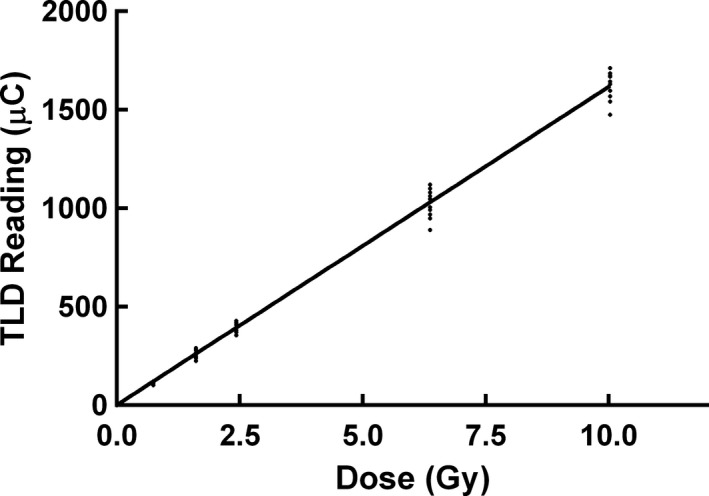
The dose–response of the TLDs below 10 Gy.

**Figure 4:**
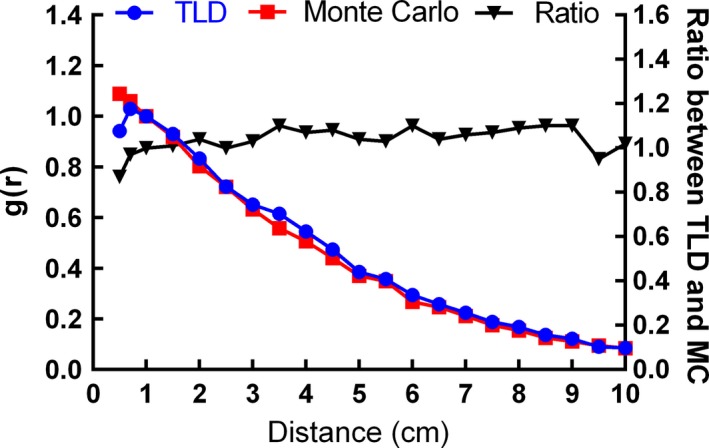
Comparison of radial dose function values derived from the Monte Carlo simulations and the experimental measurements for the GMS BT‐125‐1 ^125^I seed.

**Figure 5:**
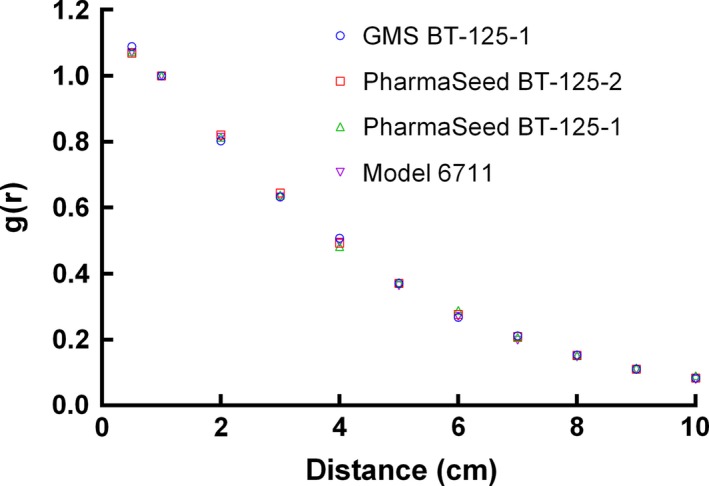
Comparison of the Monte Carlo simulated radial dose function for different radioactive seeds.

The calibration curve of TLD was shown in Fig. 3. We found the dose–response of the TLDs was linear below 10 Gy. The manufacturer‐provided seed strength of radioactive ^125^I seeds were 1.041, 1.016, 0.995, 0.991, 0.901, and 0.860 mCi, which had an uncertainty of 3% compared with the measured seed strength when using the well‐type ionization chamber (Standard Imaging, USA). Compared with the results of Model 6711 ^125^I seed provided by TG‐43U1^16^, statistical Monte Carlo uncertainties were less than 2% along the seed perpendicular axis, 0.5% at 1 cm, within 3% for polar angles 5° < θ < 90°, and within 5% for polar angles θ < 5°. The Monte Carlo simulated dose rate constant of GMS BT‐125‐1 ^125^I seed was 0.975 $cGy\cdot h^{-1}\cdot U^{-1}$ when using water phantom. The experiment measured dose rate constant of GMS BT‐125‐1 ^125^I seed was 0.943 $cGy\cdot h^{-1}\cdot U^{-1}$ after conversion from PMMA phantom to water phantom. The average of the Monte Carlo simulated, and the experimental dose rate constants was 0.959 $cGy\cdot h^{-1}\cdot U^{-1}$. Table 3 presents the dose rate constants for the GMS BT‐125‐1 ^125^I seed and other three commercially available radioactive seeds.^16^, ^27^, ^28^ The differences in dose rate constants were 0.94%, 0.83%, and 0.73% when comparing the GMS BT‐125‐1 ^125^I seed with PharmaSeed BT‐125‐1, PharmaSeed BT‐125‐2 and Model 6711 ^125^I seeds, respectively.^16^, ^27^, ^28^ The radial dose function, $g\left(r\right)$ accounted for dose fall‐off on the transverse plane due to photon scattering and attenuation. It can also be influenced by the material and encapsulation of the seed. Figure 4 presented the radial dose function comparison of the GMS BT‐125‐1 ^125^I seed between the experiment and the Monte Carlo results. The differences between the Monte Carlo and the experimental $g\left(r\right)$ results were mostly within 10%. The difference was 14.5% when *r* = 0.5 cm. The comparisons of radial dose function for different seeds using Monte Carlo were presented in Fig. 5. Monte Carlo results of $g\left(r\right)$ for GMS BT‐125‐1 ^125^I seed were found in agreement (within 3.3%) with corresponding results for the PharmaSeed BT‐125‐2 ^125^I seed.^29^

**Table 3:** Comparison of dose rate constant for different commercially available radioactive seeds

Authors	^125^I seed	Material of marker	Length of marker (cm)	Dose rate constant, ($cGy\cdot h^{-1}\cdot U^{-1}$)	Differences between the dose rate constants (%)
Popescu et al	PharmaSeed BT‐125‐1	Molybdenum rod	0.325	0.950	0.94
DeMarco et al	PharmaSeed BT‐125‐2	Silver rod	0.325	0.967	0.83
TG43U1	6711	Silver rod	0.300	0.965	0.62
This study	GMS BT‐125‐1	Silver rod	0.325	0.959	0

For the Monte Carlo simulated radial dose functions, the coefficients of the fit for GMS BT‐125‐1 ^125^I seed were a_0_ = 1.185, a_1_ = −0.175, a_2_ = −1.702E‐02, a_3_ = 6.741E‐03, a_4_ = −6.827E‐04, and a_5_ = 2.437E‐05. The Monte Carlo simulations and the experimental measurements of F(r,θ) for GMS BT‐125‐1 ^125^I seed are shown in Tables 4 and 5. For *r* > 0.5 cm, θ ≥ 20° the observed differences between the Monte Carlo and the experimental results were typically <10%. For θ < 20° or *r* = 0.5 cm, the differences were larger than 10%. Similar to the radial dose function, the polynomial of the Monte Carlo two‐dimensional anisotropy function was $f\left(\theta \right)=p_{0}+p_{1}\theta +p_{2}\theta^{2}+p_{3}\theta^{3}+p_{4}\theta^{4}+p_{5}\theta^{5}$. The coefficients of the polynomial at different distances are shown in Table 6.

**Table 4:** The Monte Carlo simulations of anisotropy functions *F*(*r*, θ) for the GMS BT‐125‐1 ^125^I seed

Distance r (cm)										
Angle(°)	0.5	0.7	1	1.5	2	3	4	5	6	7
0		0.303	0.375	0.419	0.437	0.539	0.623	0.607	0.639	0.710
10	0.387	0.450	0.512	0.590	0.574	0.639	0.729	0.693	0.666	0.758
20		0.674	0.710	0.771	0.666	0.712	0.843	0.782	0.721	0.798
30	0.778	0.809	0.810	0.809	0.705	0.807	0.912	0.850	0.837	0.873
40		0.891	0.889	0.942	0.841	0.875	0.963	0.915	0.858	0.920
50	0.906	0.949	0.966	1.031	0.931	0.988	1.029	0.947	0.917	0.957
60		1.005	0.981	1.041	0.951	0.990	1.049	0.956	0.953	1.006
70	0.949	0.991	1.015	1.053	0.960	1.028	1.045	0.985	0.941	1.013
80		0.994	0.978	1.061	1.020	1.044	1.047	1.010	0.948	0.996
90	1.000	1.000	1.000	1.000	1.000	1.000	1.000	1.000	1.000	1.000

**Table 5:** The experiment measurements of anisotropy functions of the GMS BT‐125‐1 ^125^I seed

Distance r (cm)										
Angle(°)	0.5	0.7	1	1.5	2	3	4	5	6	7
0			0.626	0.627	0.613	0.617	0.629	0.621	0.738	0.741
10	0.787	0.656	0.671	0.696	0.657	0.719	0.697	0.657	0.774	0.801
20			0.804	0.825	0.788	0.802	0.792	0.754	0.765	0.823
30	0.943	0.878	0.849	0.836	0.827	0.848	0.821	0.827	0.824	0.842
40			0.974	0.947	0.940	0.856	0.853	0.864	0.899	0.926
50	1.048	0.995	0.973	0.939	0.943	0.899	0.903	0.895	0.910	0.943
60			1.028	1.012	0.985	0.953	0.912	0.916	1.023	0.987
70	1.055	0.993	0.987	1.010	1.018	1.010	0.968	0.902	1.028	0.988
80			1.020	1.004	0.987	0.970	0.982	0.920	0.979	0.963
90	1.000	1.000	1.000	1.000	1.000	1.000	1.000	1.000	1.000	1.000

**Table 6:** Polynomial coefficients of anisotropy function for Monte Carlo at different distances

r (cm)	0.7	1	1.5	2	3	4	5	6	7
p_0_	0.298	0.370	0.417	0.439	0.541	0.622	0.608	0.639	0.709
p_1_	0.789	0.899	1.259	0.641	0.527	0.683	0.343	0.067	0.271
p_2_	1.604	0.332	1.385	0.745	0.111	0.140	1.071	1.458	0.017
p_3_	3.442	1.129	1.477	2.160	0.285	0.298	2.306	1.558	0.083
p_4_	2.217	0.610	0.934	1.791	0.331	0.246	1.648	0.429	0.142
p_5_	0.487	0.099	0.208	0.504	0.086	0.072	0.406	0.031	0.038

## 4 DISCUSSION

This is the first study to characterize the dosimetric parameters of the BT‐125‐1 ^125^I seed manufactured by GMS Pharmaceutical Company of China. In this study, we used MCNP5 to calculate the dosimetric parameters for the GMS BT‐125‐1 ^125^I seed, including dose rate constant, radial dose function, and two‐dimensional anisotropy function. The dose rate constant is used to convert the relative dose distribution into the absolute dose given to the patients, which will determine the clinical outcome. The Monte Carlo simulated and experiment measured dose rate constant of the GMS BT‐125‐1 ^125^I seed were 0.975 $cGy\cdot h^{-1}\cdot U^{-1}$ and 0.943 $cGy\cdot h^{-1}\cdot U^{-1}$, respectively. The difference between the Monte Carlo simulation and the experimental measurement was due to the uncertainty of the experimental measurement such as limited precision of repeated readings and spatial resolution, and uncertainty of the Monte Carlo simulation that was affected by the geometric uncertainty and internal component mobility. According to the TG43U1 report, we recommended the average of experimental and Monte Carlo results as the dose rate constant, which is 0.959 $cGy\cdot h^{-1}\cdot U^{-1}$.

Differences in dose rate constant were within 1% when compared to the other three types of seeds. The dose rate constants varied with different coating thicknesses, coating mass densities, photon interaction cross‐section libraries, and photon emission spectrum types.^16^, ^26^ Aryal P et al^30^ found that varying ^125^I coating thickness, coating mass density, photon cross‐section library, and photon emission spectrum for the model IA125I A^125^I seed changed the dose rate constant by up to 0.9%, about 1%, about 3%, and 3%, respectively, in comparison to the proposed standard value of 0.922 $cGy\cdot h^{-1}\cdot U^{-1}$. Due to the inverse square law, the radial dose function decreases as the distance from the transverse plane increases. Aryal P et al^30^ found that for the Monte Carlo simulation the variables influencing the radial dose function and two‐dimensional anisotropy function included spectrum, coating thickness, coating density, thickness and shape of end welding, and cross‐section library. The differences in the Monte Carlo and experimental *g*(*r*) results were mostly within 10%. The largest difference was 14.5% when *r* = 0.5 cm. The uncertainty of the experimental method became larger when the measured distance from the seed was small owing to the high gradient dose distribution near the radioactive seed. The Monte Carlo results of *g*(*r*) for the GMS BT‐125‐1 ^125^I seed were found in agreement (within 3.3%) with corresponding results for the PharmaSeed BT‐125‐2 ^125^I seed.^29^ However, it is different from PharmaSeed BT‐125‐1^125^I seed and Model 6711 ^125^I seed due to the different structures and materials of the seeds used in the Monte Carlo simulation.

For $F\left(r,\theta \right)$, biggest difference between the experimental and the Monte Carlo simulation occurs when $\theta <20^{\circ }$. It may be due to the accurate simulation of the shape of the end weld, which is relevant for two‐dimensional anisotropy function values at small angles. As the distance increases from 0.5 cm, the Monte Carlo simulated function values agree better with the experimental measurements. The differences between the measured and the Monte Carlo simulated $F\left(r,\theta \right)$ were seen to increase with decreasing the polar angle. These differences can be mainly attributed to the accuracy of the Seed/TLD positioning, the simulation of the end weld thickness, seed design, and distribution of radioactivity within the seed. The differences in Monte Carlo simulated between the GMS BT‐125‐1 ^125^I seed and other commercial seeds were due to the different material and length of the markers and different shape of end welding.

Due to the limitation of the geometry of the phantom, the uncertainty of the TLD source position is about 3.5%, which is 4.0% in TG43U1 report.^16^ The accuracy of the phantom was compromised by enlarging the hole of TLDs by 0.1 mm to load and remove the TLDs smoothly during the experiment. It is difficult for operation if the size of the holes of the phantom is the same as that of the TLD dosimeters.

Based on the study conducted by Safigholi et al,^31^ the interseed attenuation effect in multisource implant using Monte Carlo simulation was less than 5%. The interseed attenuation effect should be considered in the clinical dose calculation.

## 5 CONCLUSIONS

This is the first study to determine GMS BT‐125‐1 ^125^I seed using both the experimental measurements and Monte Carlo simulation. The measured dose rate constant, radial dose functions and two‐dimensional anisotropy functions for the GMS BT‐125‐1 ^125^I seed showed good agreement with the Monte Carlo results. The dose rate constant of the GMS BT‐125‐1 ^125^I seed is similar to that of the PharmaSeed BT‐125‐1 ^125^I seed, the PharmaSeed BT‐125‐2 ^125^I seed, and the Model 6711 ^125^I seed. For radial dose functions and two‐dimensional anisotropy functions, the GMS BT‐125‐1 ^125^I seed is similar to the PharmaSeed BT‐125‐2 ^125^I seed but different from the PharmaSeed BT‐125‐1 ^125^I seed and the Model 6711 ^125^I seed. This study improved the accuracy of dose calculation using GMS BT‐125‐1 ^125^I seed during seed implantation.

## CONFLICT OF INTEREST

The authors have no conflicts of interest to disclose.
